# Association between number of medications and indicators of potentially inappropriate polypharmacy: a population-based cohort of older adults in Quebec, Canada

**DOI:** 10.1177/20420986241309882

**Published:** 2024-12-25

**Authors:** Alexandre Campeau Calfat, Justin P. Turner, Marc Simard, Véronique Boiteau, Caroline Sirois

**Affiliations:** Faculty of Pharmacy, Université Laval, Québec, QC, Canada; Institut national de santé publique du Québec, Québec, QC, Canada; VITAM – Centre de recherche en santé durable, Québec, QC, Canada; Faculty of Pharmacy, Université Laval, Québec, QC, Canada; Centre for Medicine Use and Safety, Faculty of Pharmacy and Pharmaceutical Science, Monash University, Melbourne, VIC, Australia; Institut national de santé publique du Québec, Québec, QC, Canada; Institut national de santé publique du Québec, Québec, QC, Canada; Faculty of Pharmacy, Université Laval, CEVQ, 1050 Chemin Ste-Foy, Quebec, QC G1S 4L8, Canada; Institut national de santé publique du Québec, Québec, QC, Canada; VITAM – Centre de recherche en santé durable, Québec, QC, Canada

**Keywords:** appropriateness, medication use, older adults, polypharmacy

## Abstract

**Background:**

As the number of medications increases, the appropriateness of polypharmacy may become questionable due to the heightened risk of medication-related harm.

**Objectives:**

(1) To investigate the relationship between the number of current medications used by older adults and three indicators of potentially inappropriate polypharmacy: (a) the mean number of potentially inappropriate medications (PIMs), (b) the average count of drug–drug interactions, and (c) the anticholinergic burden; (2) To characterize the population-based burden of potentially inappropriate polypharmacy by calculating the proportion of individuals with these indicators.

**Design:**

We conducted a population-based observational study using the Quebec Integrated Chronic Disease Surveillance System.

**Methods:**

We included all individuals over 65 years insured by the public drug plan on April 1st, 2022. For each individual, we calculated the number of current medications and the number of (a) PIMs (Beers 2019), (b) drug–drug interactions (Beers 2019), and (c) anticholinergic burden (Anticholinergic Cognitive Burden (ACB) scale). The association between the number of medications and these indicators was quantified using linear regression. Prevalence with 99% confidence intervals (CIs) was calculated.

**Results:**

A total of 1,437,558 individuals (mean age: 75; 55% female) were included, taking an average of 4.9 medications (±4.1). For each additional medication, the mean number of PIMs, drug–drug interactions, and anticholinergic burden increased by 0.11, 0.04, and 0.17, respectively (*p*-trend <0.0001). Nearly half the population (45.5%; 99% CI: 45.5–45.5) had a regimen containing ⩾1 PIMs, ⩾1 drug–drug interaction, or an ACB ⩾3.

**Conclusion:**

The strong association between the increasing number of medications and reduced polypharmacy quality underscores the importance of medication count beyond therapeutic indications. With widespread medication use, many older adults face quality issues.

## Plain language summary

What is the link between number of medications and inappropriate medication use in older adults in Quebec, Canada

*Study Overview*: Taking multiple medications can increase the risk of harmful effects, especially for older adults. Our study examined the relationship between the number of medications taken and three specific risks: 1. Use of medications that may be inappropriate for older adults. 2. Presence of drug-drug interactions. 3. Use of medications that may impair cognitive function. We analyzed medications used by individuals aged 65 and over in the province of Quebec, Canada, on April 1st, 2022. *Key Findings*: As the number of medications increased, so did the likelihood of encountering at least one of these risks. Nearly half of the older adult population faced one or more of these risks. *Implications for Care*: The more medications an older adult takes, the higher the chance of reduced medication safety. This underscores the need to regularly reviewing the number of medications and assessing whether each one is necessary. Many older adults are at risk due to common practice of taking multiple medications.

## Introduction

With the aging population, the use of multiple medications, known as polypharmacy, has become increasingly prevalent.^1^ In the province of Quebec, Canada, a third of community-dwelling older adults were using 10 or more prescribed medications in 2022.^1^ The World Health Organization has outlined new goals aimed at ensuring optimal medication use by 2030.^2^ However, even when polypharmacy is deemed appropriate for managing all existing conditions, it can be challenging to ensure that its benefits outweigh its risks.^3^ As the number of medications increases, so does the risk of medication-related problems. Potential harm may arise from the use of medications whose associated risks outweigh the benefits. Such potentially inappropriate medications (PIMs) are populated in lists developed by recognized experts. The Beers criteria, for example, notably contains 30 medications or medication classes to be avoided in older adults.^4^ It is very common for older adults to use PIMs; for instance, in 2022, half of community-dwelling older adults in Quebec had at least one of such PIMs.^1^ In addition, drug–drug interactions can lead to adverse outcomes. Drug–drug interactions that should be avoided in older adults are similarly listed in the Beers criteria.^4^ Harm can further result from the anticholinergic burden, which represents the cumulative anticholinergic effect of a medication regimen. Increased anticholinergic burden has been associated with a heightened risk of cognitive impairment, among other adverse effects.^5^

Studies have demonstrated that polypharmacy is the strongest predictor of PIM use,^6,7^ and that it is also associated with drug–drug interactions,^8^ and an increase in anticholinergic burden.^9^ Therefore, the quality of a medication regimen may be compromised by the high number of medications,^10^ even when theoretically required by an individual’s conditions. Although polypharmacy is associated with PIMs, drug–drug interactions, and anticholinergic burden, to our knowledge, no study has empirically quantified these associations on a population-wide scale. Establishing a connection between the number of medications and the quality of a medication regimen could assist healthcare providers and public health efforts in identifying and addressing potentially at-risk polypharmacy cases. This approach could aid in distinguishing appropriate from potentially inappropriate polypharmacy, a recognized need.^3,11–13^

The overarching aim of our study was to characterize the association between quality and quantity of medications, beyond clinical characteristics and therapeutic indications. Our specific objectives were (1) to investigate the relationship between the number of medications used by older adults and three indicators of potentially inappropriate polypharmacy: (a) the mean number of PIMs, (b) the average count of drug–drug interactions, and (c) the anticholinergic burden; (2) to characterize the population-based burden of potentially inappropriate polypharmacy by calculating the proportion of individuals with these indicators.

## Methods

### Study design and data source

We conducted a population-based study using the Quebec Integrated Chronic Disease Surveillance System (QICDSS), managed by the National Public Health Institute of the province of Quebec, Canada (*Institut national de santé publique du Québec* (INSPQ)). The reporting of this study conforms to the STROBE statement.^14^

With data available since 1996, the QICDSS combines, under a unique anonymized identification number, information from five databases: (1) the health insurance registry, which contains sociodemographic data; (2) the MED-ÉCHO database, which contains hospitalization data; (3) the vital statistics death database, which contains the date of death and associated causes; (4) the physician claims database, which contains data on all fee-for-service billings, including diagnostic codes; (5) the pharmaceutical services database, which contains data on prescription medication claims under the public drug plan. The public health plan, which encompasses hospitalization and medical care coverage, covers approximately 99% of the province’s population.^15^ Furthermore, about 90% of individuals aged 65 years and older are covered by the public drug plan, which specifically addresses medication coverage.^15^

### Study population

We identified all community-dwelling individuals aged over 65 years who were covered by the public drug plan on April 1st, 2022. Individuals had to be covered by the public health plan throughout 2022 and by the public drug plan from October 1st, 2021, to April 1st, 2022. We chose a 6-month lookback period to consider medications with infrequent dispensing (e.g., denosumab). We excluded individuals residing in long-term care facilities because information on their medication use is not included in the QICDSS.

### Current medications on April 1st, 2022

The common denomination code (equivalent to the fifth level of the Anatomical Therapeutic Chemical (ATC) classification) was used to ascertain medications in the pharmaceutical services database of the QICDSS. Medications with multiple active ingredients were classified under one code (e.g., a tablet with two active ingredients was counted as one medication). For a medication to be considered, its duration of treatment, as recorded by the pharmacist at the time of dispensing, had to include the date of April 1st, 2022 (Supplemental Figure S1). As-needed medications were also included under the same condition. All types of medications were considered (oral, topical, inhaled, etc.). Medications not covered by the public drug plan (e.g., over-the-counter medications, natural products) are not included in the database and therefore could not be assessed. The number of medications taken by each individual on April 1st, 2022, was calculated. We created categories for each number of medications (1, 2, . . ., ⩾20). For the analyses, we combined users of ⩾20 medications as they represented less than 0.5% of the population. The number of medications in 1 day represents the clinical situation where an individual presents themselves to a health professional with their medication list.

### PIMs, drug–drug interactions, and anticholinergic burden

PIMs were identified using the 2019 update of the American Geriatrics Society Beers Criteria.^4^ These criteria are widely used in studies conducted with administrative data because they require limited clinical information.^16–18^ The list was adapted to align with available medications in Canada and data in the QICDSS.^16^ Any medication listed in Table 2 of the Beers criteria was considered a PIM. However, doxepin and digoxin were excluded as PIMs since dosage information was not considered in our study. Aspirin was classified as a PIM if it was used for primary prevention, according to the 2023 update of the Beers criteria.^19^ Diagnostic codes related to coronary events, heart failure, and cerebral or vascular diseases were used to ascertain the indication of aspirin in secondary prevention. This approach was unique to aspirin because the change in recommendation for aspirin use was significant between 2019 and 2023.^4,19^ Aspirin’s role in primary prevention has been subject to extensive new research and guideline updates,^19^ making it a critical exception. The total number of PIMs for each individual was calculated, with each medication counted once, even if it appeared multiple times in the Beers criteria. We further categorized individuals into two groups: no PIMs and ⩾1 PIMs.

Drug–drug interactions were identified using the 2019 update of the American Geriatrics Society Beers Criteria which lists “potentially clinically important drug–drug interactions that should be avoided in older adults.”^4^ A combination of medications listed in Table 5 of the Beers criteria was considered as a drug–drug interaction. The total number of drug–drug interactions for each individual was calculated. We further categorized individuals into two groups: no drug–drug interactions and ⩾1 drug–drug interactions.

The anticholinergic burden was measured using the Anticholinergic Cognitive Burden (ACB) scale. This validated scale has been used to measure risk of cognitive decline in older adults.^20,21^ The anticholinergic burden score was assigned to each medication (with ACB levels ranging from 0 to 3),^5^ and the scores were subsequently summed for each individual according to the medications they were taking. We further categorized ACB levels into three dichotomous groups: ACB level ⩾1 (including ACB levels ⩾2 and ⩾3, compared to ACB level 0), ACB level ⩾2 (including ACB level ⩾3, compared to ACB levels 0 and 1), and ACB level ⩾3 (compared to ACB levels 0, 1, and 2). Higher ACB levels indicate a greater anticholinergic burden and an increased risk of cognitive impairment, among other negative consequences.^5^

### Other variables

Age, sex, social and material deprivation index, living zone (urban vs rural), and the presence of chronic diseases were used exclusively to characterize the studied population and were not incorporated into subsequent analyses, aligning with the objective to examine pharmacotherapy independently of the patient’s characteristics. Deprivation indexes are validated proxies of socioeconomic status based on postal code.^22^ The quality of social networks can be expressed by the social deprivation index, whereas the quality of material goods owned by the population studied can be gauged by the material deprivation index. The first quintile of both indexes includes the least deprived individuals while the fifth quintile contains the most deprived.^22^ We used validated definitions, that is standardized, evidence-based set of criteria based notably on diagnosis codes and procedures, to accurately identify individuals with the following chronic diseases: asthma, chronic obstructive pulmonary disease, dementia, diabetes, hypertension, mood disorders, osteoporosis, schizophrenia, and stroke.^15^ In addition, we calculated the comorbidity score, considering the 31 diseases included in the combined comorbidity score, which has been validated for the Quebec population.^23^ The comorbidity score predicts the impact of chronic diseases on mortality, with an increase in risk associated with a higher score.^23^

### Statistical analyses

All individuals of the population, and all individuals with at least one medication on April 1st, 2022, were characterized with descriptive statistics. We studied the entire population, not just those with medications, to assess the public health impact of potentially inappropriate polypharmacy. While focusing on individuals with medications is clinically valid and relevant, examining the broader population highlights the potential for substantial public health benefits from population-wide interventions.

The mean number of PIMs, of drug–drug interactions, and the mean anticholinergic burden score, with their 99% confidence intervals (CIs), were calculated for each number of current medications. We used 99% CI, which is a stricter threshold than the conventional 95%, because our large population provides very high statistical power, allowing even small differences to be statistically significant. The association between the number of current medications and (1) mean number of PIMs, (2) mean number of drug–drug interactions, and (3) mean anticholinergic burden score was observed graphically to facilitate the visualization and interpretation of the relationship between the number of current medications and those continuous variables.^24,25^ Because the relationship was mostly linear, we conducted three separate univariate linear regressions to assess the association (trend) between the number of current medications (treated as a continuous variable) and each of the following: the mean number of PIMs, drug–drug interactions, and the ACB score.

For each group of individuals with the same number of current medications, we calculated the proportion of individuals with (1) at least one PIM, (2) at least one drug–drug interaction, (3) an anticholinergic burden of ⩾1, ⩾2 and ⩾3. In addition, we determined the proportion of individuals with potentially inappropriate polypharmacy, defined as having at least one of the three indicators (PIM, drug–drug interaction, or anticholinergic burden) for each group with the same number of current medications. From a public health perspective, to estimate the burden of potentially inappropriate polypharmacy in the population, we calculated the proportion of individuals exposed to one of the three indicators (PIMs, drug–drug interactions, anticholinergic burden) using the entire population as the denominator. We also calculated those proportions using the number of individuals with at least X number of current medications as the denominator.

Sensitivity analyses were conducted to validate the robustness of the association between the number of current medications and the three indicators. We used 2019 data for temporal validation, as it is the most recent year before the pandemic and the year of the last update of the Beers criteria before our study period.^4^

All analyses were conducted using SAS version 9.4 (SAS Institute, Cary, NC, USA).

### Ethics

The provincial Public Health Research Ethics Board and the Quebec Commission protecting access to information have approved the use of the QICDSS for surveillance purposes. No written informed consent was required. The ethics board of the *Centre intégré universitaire de santé et de services sociaux de la Capitale-Nationale* confirmed that this project is exempt from ethics approval (project #2023-2793).

## Results

There were 1,665,850 community-dwelling individuals aged over 65 years on April 1st, 2022. We excluded 228,292 individuals who were not covered by the public health plan throughout 2022 or by the public drug plan from October 1st, 2021, to April 1st, 2022. Thus, our study included 1,437,558 individuals (mean age 75.5 years, 54.8% female, mean number of medications 4.9 ± 4.1; Table 1). From them, 1,214,195 (84.5%) had at least one medication on April 1st, 2022 (mean: 5.8 ± 3.8 medications; Table 2). Most medication users (55.9%) had at least five current medications. More than 4 in 10 individuals in the population (43.9%) had at least one PIM, 6.0% had at least one drug–drug interaction, and 29.5% had an ACB level of one or higher.

**Table 1.** Characteristics of community-dwelling adults aged over 65 years insured by the public drug plan in Quebec, Canada, on April 1st, 2022.

Characteristics	All individuals		Individuals with at least one medication on April 1st, 2022	
	*N* = 1,437,558		*N* = 1,214,195	
	*n*	%	*n*	%
Age, years (mean ± SD)	75.5 ± 7.0		76.0 ± 7.1	
Age, years				
66–70	460,937	32.1	355,356	29.3
71–75	392,755	27.3	330,874	27.3
76–80	279,980	19.5	247,961	20.4
81–85	166,875	11.6	152,520	12.6
⩾86	137,011	9.5	127,484	10.5
Sex				
Female	787,346	54.8	677,935	55.8
Male	650,212	45.2	536,260	44.2
Social deprivation index				
1 (least deprived)	244,741	17.0	204,316	16.8
2	265,097	18.4	223,331	18.4
3	264,040	18.4	223,069	18.4
4	269,571	18.8	226,834	18.7
5 (most deprived)	259,514	18.1	217,300	17.9
Unknown	134,595	9.4	119,345	9.8
Material deprivation index				
1 (least deprived)	241,674	16.8	198,601	16.4
2	245,567	17.1	206,439	17.0
3	259,104	18.0	219,267	18.1
4	275,268	19.2	233,825	19.3
5 (most deprived)	281,350	19.6	236,718	19.5
Unknown	134,595	9.4	119,345	9.8
Zone				
Urban (10k inhabitants or more)	1,121,848	78.0	946,965	78.0
Rural (less than 10k inhabitants)	313,876	21.8	265,994	21.9
Unknown	1834	0.1	1236	0.1
Combined comorbidity score, mean ± SD	1.6 ± 2.6		1.7 ± 2.7	
Comorbidities*				
Asthma	156,310	10.9	142,540	11.7
Chronic obstructive pulmonary disease	263,893	18.4	241,844	19.9
Dementia	60,540	4.2	57,467	4.7
Diabetes	334,775	23.3	313,017	25.8
Hypertension	836,163	58.2	775,331	63.9
Mood disorder	96,337	6.7	88,626	7.3
Osteoporosis	372,897	25.9	334,481	27.6
Schizophrenia	3736	0.3	3504	0.3
Stroke	108,939	7.6	101,483	8.4

* Presence of individual comorbidities using the validated case definitions of the Quebec Integrated Chronic Disease Surveillance System.

**Table 2.** Number of current medications, potentially inappropriate medications, drug–drug interactions, and anticholinergic burden level in community-dwelling adults aged over 65 years insured by the public drug plan in Quebec, Canada, on April 1st, 2022.

Medication characteristics	All individuals		Individuals with at least one medication on April 1st, 2022	
	*N* = 1,437,558		*N* = 1,214,195	
	*n*	%	*n*	%
Number of current medications, mean ± SD	4.9 ± 4.1		5.8 ± 3.8	
Number of current medications* (at least X current medications)				
0	223,363	15.5	NA	NA
1	116,750	8.1	116,750	9.6
(⩾1)	(1,214,195)	(84.5)	(1,214,195)	(100)
2	135,010	9.4	135,010	11.1
(⩾2)	(1,097,445)	(76.3)	(1,097,445)	(90.5)
3	143,818	10.0	143,818	11.8
(⩾3)	(962,435)	(66.9)	(962,435)	(79.4)
4	141,855	9.9	141,855	11.7
(⩾4)	(818,617)	(56.9)	(818,617)	(67.6)
5	131,631	9.2	131,631	10.8
(⩾5)	(676,762)	(47.1)	(676,762)	(55.9)
6	115,329	8.0	115,329	9.5
(⩾6)	(545,131)	(37.9)	(545,131)	(45.1)
7	97,810	6.8	97,810	8.1
(⩾7)	(429,802)	(29.9)	(429,802)	(35.6)
8	79,777	5.5	79,777	6.6
(⩾8)	(331,992)	(23.1)	(331,992)	(27.5)
9	63,563	4.4	63,563	5.2
(⩾9)	(252,215)	(17.5)	(252,215)	(20.9)
10	50,351	3.5	50,351	4.2
(⩾10)	(188,652)	(13.1)	(188,652)	(15.7)
11	38,663	2.7	38,663	3.2
(⩾11)	(138,301)	(9.6)	(138,301)	(11.5)
12	28,866	2.0	28,866	2.4
(⩾12)	(99,638)	(6.9)	(99,638)	(8.3)
13	21,234	1.5	21,234	1.8
(⩾13)	(70,772)	(4.9)	(70,772)	(5.9)
14	15,243	1.1	15,243	1.3
(⩾14)	(49,538)	(3.4)	(49,538)	(4.1)
15	11,169	0.8	11,169	0.9
(⩾15)	(34,295)	(2.4)	(34,295)	(2.8)
16	7735	0.5	7735	0.6
(⩾16)	(23,126)	(1.6)	(23,126)	(1.9)
17	5234	0.4	5234	0.4
(⩾17)	(15,391)	(1.1)	(15,391)	(1.3)
18	3577	0.2	3577	0.3
(⩾18)	(10,157)	(0.7)	(10,157)	(0.9)
19	2390	0.2	2390	0.2
(⩾19)	(6580)	(0.5)	(6580)	(0.6)
⩾20	4190	0.3	4190	0.4
Number of potentially inappropriate medications, mean ± SD	0.6 ± 0.8		0.7 ± 0.8	
Number of potentially inappropriate medications (at least X PIMs)				
0	807,145	56.1	583,782	48.1
1	476,136	33.1	476,136	39.2
(⩾1)	(630,413)	(43.9)	(630,413)	(52.0)
2	123,337	8.6	123,337	10.2
(⩾2)	(154,277)	(10.8)	(154,277)	(12.8)
3	25,351	1.8	25,351	2.1
(⩾3)	(30,940)	(2.2)	(30,940)	(2.6)
4	4740	0.3	4740	0.4
(⩾4)	(5589)	(0.4)	(5589)	(0.5)
5	730	0.1	730	0.1
(⩾5)	(849)	(0.1)	(849)	
6	108	0.0	108	0.0
(⩾6)	(119)		(119)	
⩾7	11	0.0	11	0.0
Number of drug–drug interactions, mean ± SD	0.1 ± 0.4		0.1 ± 0.4	
Number of drug–drug interactions (at least X drug–drug interactions)				
0	1,351,092	94.0	1,127,729	92.9
1	70,553	4.9	70,553	5.8
(⩾1)	(86,466)	(6.0)	(86,466)	(7.1)
2	11,302	0.8	11,302	0.9
(⩾2)	(15,913)	(1.1)	(15,913)	(1.3)
3	4208	0.3	4208	0.4
(⩾3)	(4611)	(0.3)	(4611)	(0.4)
4	388	0.0	388	0.0
(⩾4)	(403)		(403)	
⩾5	15	0.0	15	0.0
Level of anticholinergic burden, mean ± SD	0.5 ± 0.7		0.7 ± 1.3	
Level of anticholinergic burden (at least X ACB level)				
0	1,013,519	70.5	790,156	65.1
1	218,474	15.2	218,474	18.0
(⩾1)	(424,039)	(29.5)	(424,039)	(34.8)
2	61,784	4.3	61,784	5.1
(⩾2)	(205,565)	(14.3)	(205,565)	(16.8)
3	81,115	5.6	81,115	6.7
(⩾3)	(143,781)	(10.0)	(143,781)	(11.7)
4	34,290	2.4	34,290	2.8
(⩾4)	(62,666)	(4.4)	(62,666)	(5.0)
5	12,265	0.9	12,265	1.0
(⩾5)	(28,376)	(2.0)	(28,376)	(2.2)
6	8627	0.6	8627	0.7
(⩾6)	(16,111)	(1.1)	(16,111)	(1.2)
7	4126	0.3	4126	0.3
(⩾7)	(7484)	(0.5)	(7484)	(0.5)
8	1664	0.1	1664	0.1
(⩾8)	(3358)	(0.2)	(3358)	(0.2)
9	965	0.1	965	0.1
(⩾9)	(1694)	(0.1)	(1694)	(0.1)
10	427	0.0	427	0.0
(⩾10)	(729)		(729)	
11	171	0.0	171	0.0
(⩾11)	(302)		(302)	
12	69	0.0	69	0.0
(⩾12)	(131)		(131)	
13	44	0.0	44	0.0
(⩾13)	(62)		(62)	
14	11	0.0	11	0.0
(⩾14)	(18)		(18)	
⩾15	7	0.0	7	0.0

* Since our study focused on a single day, some individuals may not have been recorded as using medications on that specific day, despite actually being on treatment. This could include individuals who were late in renewing their prescriptions or those who were previously hospitalized, where medications might have been provided by the hospital while they still had prescriptions at home. In addition, some individuals with chronic conditions may be managed through non-pharmacological treatments, particularly among the youngest segment of our older population. Finally, those who are at the end of life may not be using medications.

ACB, Anticholinergic Cognitive Burden.

**Figure 1.**
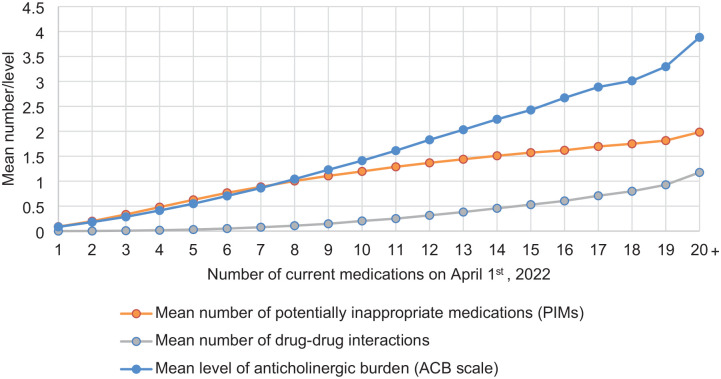
Association between potentially inappropriate medications, drug–drug interactions, anticholinergic burden, and the number of medications in community-dwelling medication users aged over 65 years in Quebec, Canada, on April 1st, 2022. Equations: Mean number of PIMs = 0.02877 + 0.11296 (number of current medications); (*p*-trend <0.0001); Mean number drug–drug interactions = −0.11469 + 0.03539 (number of current medications); (*p*-trend<0.0001); Mean level of ACB = −0.22463 + 0.16746 (number of current medications); (*p*-trend <0.0001).

The relationship between the number of current medications used by older adults and the mean number of PIMs, the average count of drug–drug interactions, and the anticholinergic burden is presented graphically in Figure 1. The presence of PIMs, drug–drug interactions, and anticholinergic burden increased mostly linearly with the number of current medications (Figure 1; Supplemental Table S1). Linear regression indicated that with each additional medication, the mean number of PIMs, drug–drug interactions, and the anticholinergic burden increased by 0.11, 0.04, and 0.17, respectively (*p*-trend <0.0001). Due to the monotonically increasing associations, it is possible to identify medication thresholds at which certain specific mean values are reached. For example, the mean number of PIMs exceeded 1.0 at a threshold of eight current medications. The mean ACB score exceeded 1, 2, and 3 at thresholds of 8, 13, and 18 current medications, respectively. By contrast, the mean number of drug–drug interactions remained lower, exceeding 1.0 at a threshold of ⩾20 current medications.

**Figure 2.**
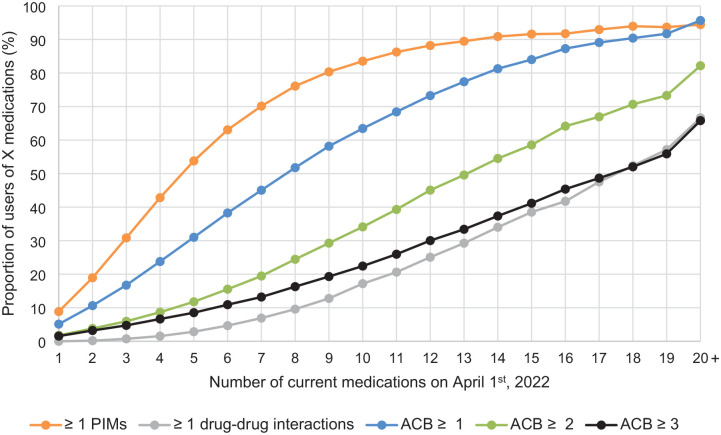
Proportions of community-dwelling medication users aged over 65 years in Quebec, Canada, with indicators of potentially inappropriate polypharmacy according to the number of current medications on April 1st, 2022. ACB, Anticholinergic Cognitive Burden; PIM, potentially inappropriate medication.

To address objective 2 from a clinical perspective, Figure 2 shows the proportions of individuals with PIMs, drug–drug interactions, and anticholinergic burden, according to the number of current medications they were taking. For example, among individuals with five current medications, more than half (53.8%) had a least one PIM, 2.9% had at least one drug–drug interaction, and 31.0% had an ACB level ⩾1. Among individuals with eight current medications, the proportions increased: 76.1% had a least one PIM, 9.6% had at least one drug–drug interaction, and 51.8% had an ACB level of one or more. Among individuals with 10 current medications, all proportions further increased: 83.5% had a least one PIM, 17.2% had at least one drug–drug interaction, and 63.5% had an ACB level of one or more.

Table 3 exposes the population-based burden of potentially inappropriate polypharmacy. On April 1st, 2022, 52.0% of the population had at least one PIM, one drug–drug interaction, or an ACB level ⩾1, and 45.5%, with an ACB ⩾3 (Table 3). Nearly 40% of the population had at least five current medications including at least one indicator of potentially inappropriate polypharmacy (Table 3). Those using at least 10 current medications represented 13.1% of the population, and those combining 10 current medications with at least one of the three indicators comprised 12.6% of the population (Table 3). The burden of potentially inappropriate polypharmacy when restricted to medication users is included in Supplemental Files (Supplemental Tables S2 and S3). For example, among individuals using at least five current medications, 83.4% had therapy with at least one indicator (Supplemental Table S3).

**Table 3.** Proportion of community-dwelling individuals aged >65 years in Quebec, Canada, with at least one PIM, one drug–drug interaction, or an anticholinergic burden according to the number of current medications on April 1st, 2022.

Number of current medications (X)	Community-dwelling individuals (*N* = 1,437,558)			
	Proportion of community-dwelling individuals using at least X medications	Proportion of community-dwelling individuals using at least X medications with at least		
		1 PIM, 1 drug–drug interaction, or an ACB level ⩾1*	1 PIM, 1 drug–drug interaction, or an ACB level ⩾2*	1 PIM, 1 drug–drug interaction, or an ACB level ⩾3*
⩾1	84.5	52.0	46.5	45.5
⩾2	76.3	51.0	45.8	44.8
⩾3	67.0	48.6	43.9	42.9
⩾4	56.9	44.6	40.6	39.7
**⩾5** **	**47.1**	**39.3**	**36.1**	**35.3**
⩾6	37.9	33.2	30.9	30.2
⩾7	29.9	27.1	25.5	25.0
⩾8	23.1	21.5	20.4	20.0
⩾9	17.5	16.7	15.9	15.6
⩾10	13.1	12.6	12.2	11.9
⩾11	9.6	9.4	9.1	8.9
⩾12	6.9	6.8	6.6	6.5
⩾13	4.9	4.9	4.8	4.7
⩾14	3.5	3.4	3.4	3.3
⩾15	2.4	2.4	2.4	2.3
⩾16	1.6	1.6	1.6	1.6
⩾17	1.1	1.1	1.1	1.1
⩾18	0.7	0.7	0.7	0.7
⩾19	0.5	0.5	0.5	0.5
⩾20	0.3	0.3	0.3	0.3

The burden of potentially inappropriate polypharmacy when restricted to medication users is presented in Supplemental Table S2.

ACB, Anticholinergic Cognitive Burden; PIM, potentially inappropriate medication.

* 99% confidence intervals are identical to the proportions presented in two decimal places. They are not shown to simplify reading.

** Example of interpretation: 39.3% of individuals in the whole population have at least five current medications and at least one indicator.

### Sensitivity analysis

Our analyses of the 2019 data showed that the association between the number of medications and the mean number of PIMs, drug–drug interactions, and ACB level is stable over time. The proportion of individuals using PIMs, drug–drug interactions, or an ACB level ⩾1, ⩾2, and ⩾3 was also stable over time (Supplemental Tables S4–S9; Supplemental Figures S2 and S3).

## Discussion

We empirically described the strong association between the increasing number of medications and the increasing presence of PIMs, drug–drug interactions, and anticholinergic burden in community-dwelling medication users aged over 65 years.

Our study is among the few that simultaneously examines the association between the number of current medications and PIMs, drug–drug interactions, and ACB burden.^10,17,26^ Moreover, it is one of the few studies to empirically quantify these associations. Several studies have highlighted the association between polypharmacy and the use of PIMs. A systematic review focusing on community-dwelling older adults in the United States showed a consistent association between polypharmacy and the use of PIMs from the Beers criteria.^7^ Similar results were also reported in Europe^27^ and China.^28^ However, these studies typically measured the risk of using at least one PIM, rather than the number of PIMs. For instance, an analysis of administrative data from 1996 in older American adults revealed that those taking more than the median annual number of medications (14) faced nearly triple the risk of having a medication from the Beers list (odds ratio = 2.9; 95% CI: 2.3–3.6) compared to those taking 14 and fewer medications.^29^ More recent data from the US Veterans database for 2015–2016 indicated that the prevalence of PIM users increased with the number of medications taken: 14.3%, 59.9%, and 83.3% among individuals using 0–4, 5–9, and ⩾10 chronic medications, respectively.^30^ Our study contributes to the literature by precisely quantifying the linear association between the number of medications used and the number of PIMs. Regarding drug–drug interactions, a similar association was exposed between simultaneous polypharmacy and drug–drug interactions in a 2005 observational study of Swedish adults aged 75 years and over (mean age of 82 (±5.3) years old).^8^ In a small Turkish study involving 420 outpatients aged 60 years and over, individuals using ⩾5 medications received a mean number of 8.1 ± 2.9 medications and had a mean ACB score of 0.99 ± 1.13.^9^ This aligns with our study showing a mean ACB score of 1.0 at a threshold of eight medications.

While the presence of PIMs, drug–drug interactions, and anticholinergic burden monotonically increase with the increasing number of current medications, some thresholds are interesting to discuss. From the threshold of five medications, more than 50% of individuals within each category of medication users were exposed to at least one PIM. The presence of PIMs was the indicator that most contributed to the prevalence of potentially inappropriate polypharmacy in our study. Anticholinergic burden also contributed to potentially inappropriate polypharmacy, with 50% of the population being exposed to an ACB level ⩾1 at a threshold of eight medications. Drug–drug interaction was the indicator that contributed less to the prevalence of potentially inappropriate polypharmacy. This may be because prescribers and pharmacists have been aware of the risks of drug–drug interactions for a long time and that several software programs offer assistance for their management.^31,32^ This could indicate that efforts to improve medication use that are only focused on reducing drug–drug interactions would miss more pressing opportunities to improve medication regimens.

The threshold of five medications is commonly used to define polypharmacy.^11,13^ Our results show that 47.1% of the population had at least five medications, and 83.4% of them had a regimen that could potentially increase medication-related harm. Thus, from a public health perspective, our results show that the traditional threshold of five medications is a reasonable cut-point for reviewing medication regimens to ensure they are appropriate. However, given the time and resource constraints associated with clinical activities in pharmacy, it may be reasonable to set a threshold higher than five medications for focusing medication review efforts on a targeted number of older adults.^33–35^ For example, individuals with at least eight medications represent a quarter of our population, among whom nearly everyone (93.2%) was at increased risk of medication-related harm.

There is a pressing need to better characterize inappropriate polypharmacy from appropriate polypharmacy.^3,11,13,36^ At a population level, the clinical definition of appropriate polypharmacy is challenging to apply, as the number and types of medications needed may vary based on an individual’s diseases, health goals, frailty status, and life expectancy perspectives.^37^ However, our results reveal a strong association between the increasing number of medications and the appropriateness of polypharmacy. Based on the three indicators, polypharmacy could be deemed potentially inappropriate once the number of current medications exceeds a specific threshold. This assessment can be conducted independently of clinical conditions or treatment goals. Such an approach offers significant advantages from a public health standpoint, especially considering the limited availability of clinical data and the life goals of patients.

### Strengths and limits

Our study has many strengths. Our population-based study gives a detailed and recent portrait of medication use in community-dwelling older adults. The analyses and variables are easy to interpret and implement in clinical contexts and for public health surveillance.

The model is not intended to explain the relationship between the variables, identify strategies to address the issue, or pinpoint factors for intervention to optimize medication use. Rather, it serves as a reminder that the quality of pharmacotherapy tends to be compromised when the number of medications is high, regardless of the underlying reasons for this increase. Our study shares the limitations of administrative databases. While we measured the dispensing of medications, we cannot guarantee their actual use. This limitation can impact levels of drug–drug interactions and anticholinergic burden, as both depend on the simultaneous use of medications. This assumption may not always hold, particularly if medications are taken only as needed. Therefore, the burden may have been overestimated in some cases. On the other hand, medication use can be underestimated by the exclusion of over-the-counter medications and medications excluded from the public insurance plan. Accordingly, the measure of all three indicators may also be underestimated. Also, PIM use was likely underestimated since we excluded medications that are PIMs according to their dosage (e.g., digoxin, doxepin). We believe this underestimation has little impact on our results because a previous study of a representative sample of Quebec’s 2017 population of individuals aged over 65 years revealed that only 0.1% and 0.2% were users of digoxin ⩾0.125 mg/day and doxepin ⩾6 mg/day, respectively.^16^ To make sure our results remain relevant to clinical practices and trends, such as updates of the Beers criteria, our analysis should be repeated periodically. Despite those limits, our sensitivity analysis indicates that the strong association between the number of medications and the studied indicators is likely to persist. Future research should investigate the risks of adverse outcomes associated with different polypharmacy thresholds.

## Conclusion

Our study confirms that the increased simultaneous medication use can lead to potential inappropriate polypharmacy. The numbers of PIMs, drug–drug interactions, and the anticholinergic burden continuously increase with the number of medications dispensed. Thus, there is a strong association between the increasing number of medications and the increasing potential for medication harm, with quality potentially compromised at traditional thresholds of polypharmacy. Our study reveals that even if all the medications are indicated, healthcare providers should carefully consider the number of medications because of the relationship between the number of medications and the potential for poor-quality outcomes.

## Supplemental Material

sj-docx-1-taw-10.1177_20420986241309882 – Supplemental material for Association between number of medications and indicators of potentially inappropriate polypharmacy: a population-based cohort of older adults in Quebec, Canada Supplemental material, sj-docx-1-taw-10.1177_20420986241309882 for Association between number of medications and indicators of potentially inappropriate polypharmacy: a population-based cohort of older adults in Quebec, Canada by Alexandre Campeau Calfat, Justin P. Turner, Marc Simard, Véronique Boiteau and Caroline Sirois in Therapeutic Advances in Drug Safety

sj-docx-2-taw-10.1177_20420986241309882 – Supplemental material for Association between number of medications and indicators of potentially inappropriate polypharmacy: a population-based cohort of older adults in Quebec, Canada Supplemental material, sj-docx-2-taw-10.1177_20420986241309882 for Association between number of medications and indicators of potentially inappropriate polypharmacy: a population-based cohort of older adults in Quebec, Canada by Alexandre Campeau Calfat, Justin P. Turner, Marc Simard, Véronique Boiteau and Caroline Sirois in Therapeutic Advances in Drug Safety

## Declarations

**Ethics approval and consent to participate:** The provincial Public Health Research Ethics Board and the Quebec Commission protecting access to information have approved the use of the QICDSS for surveillance purposes. No written informed consent was required. The ethics board of the Centre intégré universitaire de santé et de services sociaux de la Capitale-Nationale confirmed that this project is exempt from ethics approval (project #2023-2793).

**Consent for publication:** Not applicable.

**Availability of data and materials:** The datasets generated and analyzed during the current study are not publicly available due to individual privacy stakes.

## References

[1] Institut national d’excellence en santé et en services sociaux (INESSS). Portrait de la polypharmacie et de l’usage de médicaments potentiellement inappropriés chez les personnes âgées au Québec. Québec, QC: INESSS, 2024, p. 61.

[2] Mair A. Fernandez-Llimos F. , et al. Polypharmacy management by 2030: a patient safety challenge. Edinburgh: SIMPATHY Consortium, http://www.simpathy.eu/resources/publications/simpathy-project-reference-book (2017, accessed 4 September 2024).

[3] Masnoon N Shakib S Kalisch-Ellett L , et al. What is polypharmacy? A systematic review of definitions. BMC Geriatr 2017; 17(1): 230. 29017448 10.1186/s12877-017-0621-2 PMC5635569

[4] American Geriatrics Society Beers Criteria^®^ Update Expert Panel. American Geriatrics Society 2019 updated AGS Beers Criteria^®^ for potentially inappropriate medication use in older adults. J Am Geriatr Soc 2019; 67(4): 674–694. 30693946 10.1111/jgs.15767

[5] Boustani M Campbell N Munger S , et al. Impact of anticholinergics on the aging brain: a review and practical application. Aging Health 2008; 4(3): 311–320.

[6] Roux B Sirois C Simard M , et al. Potentially inappropriate medications in older adults: a population-based cohort study. Fam Pract 2020; 37(2): 173–179. 31602472 10.1093/fampra/cmz060

[7] Nothelle SK Sharma R Oakes A , et al. Factors associated with potentially inappropriate medication use in community-dwelling older adults in the United States: a systematic review. Int J Pharm Pract 2019; 27(5): 408–423. 30964225 10.1111/ijpp.12541 PMC7938818

[8] Johnell K. Klarin I. The relationship between number of drugs and potential drug-drug interactions in the elderly: a study of over 600 000 elderly patients from the Swedish Prescribed Drug Register. Drug Saf 2007; 30(10): 911–918. 17867728 10.2165/00002018-200730100-00009

[9] Koc Okudur S Dokuzlar O Aydin AE , et al. The evaluation of relationship between polypharmacy and anticholinergic burden scales. North Clin Istanb 2021; 8(2): 139–144. 33851077 10.14744/nci.2020.17136 PMC8039107

[10] Lampe D Grosser J Gensorowsky D , et al. The relationship of continuity of care, polypharmacy and medication appropriateness: a systematic review of observational studies. Drugs Aging 2023; 40(6): 473–497. 36972012 10.1007/s40266-023-01022-8 PMC10232587

[11] Sirois C Domingues NS Laroche M-L , et al. Polypharmacy definitions for multimorbid older adults need stronger foundations to guide research, clinical practice and public health. Pharmacy 2019; 7(3): 126. 31470621 10.3390/pharmacy7030126 PMC6789889

[12] Lau SR Waldorff F Holm A , et al. Disentangling concepts of inappropriate polypharmacy in old age: a scoping review. BMC Public Health 2023; 23(1): 245. 36739368 10.1186/s12889-023-15013-2 PMC9899389

[13] Guillot J Maumus-Robert S Bezin J. Polypharmacy: a general review of definitions, descriptions and determinants. Therapies 2020; 75(5): 407–416. 10.1016/j.therap.2019.10.001 31732240

[14] von Elm E Altman DG Egger M , et al. The Strengthening the Reporting of Observational Studies in Epidemiology (STROBE) statement: guidelines for reporting observational studies. J Clin Epidemiol 2008; 61(4): 344–349. 18313558 10.1016/j.jclinepi.2007.11.008

[15] Blais C Jean S Sirois C , et al. Quebec Integrated Chronic Disease Surveillance System (QICDSS), an innovative approach. Chronic Dis Inj Can 2014; 34(4): 226–235. 25408182

[16] Gosselin M Talbot D Simard M , et al. Classifying polypharmacy according to pharmacotherapeutic and clinical risks in older adults: a latent class analysis in Quebec, Canada. Drugs Aging 2023; 40(6): 573–583. 37149556 10.1007/s40266-023-01028-2

[17] Mohamed MR Ramsdale E Loh KP , et al. Associations of polypharmacy and inappropriate medications with adverse outcomes in older adults with cancer: a systematic review and meta-analysis. Oncologist 2020; 25(1): e94–e108. 10.1634/theoncologist.2019-0406 PMC6964156 31570516

[18] Mekonnen AB Redley B de Courten B , et al. Potentially inappropriate prescribing and its associations with health-related and system-related outcomes in hospitalised older adults: a systematic review and meta-analysis. Br J Clin Pharmacol 2021; 87(11): 4150–4172. 34008195 10.1111/bcp.14870 PMC8597090

[19] American Geriatrics Society Beers Criteria^®^ Update Expert Panel. American Geriatrics Society 2023 updated AGS Beers Criteria^®^ for potentially inappropriate medication use in older adults. J Am Geriatr Soc 2023; 71(7): 2052–2081. 37139824 10.1111/jgs.18372 PMC12478568

[20] Salahudeen MS Duffull SB Nishtala PS. Anticholinergic burden quantified by anticholinergic risk scales and adverse outcomes in older people: a systematic review. BMC Geriatr 2015; 15(1): 31. 25879993 10.1186/s12877-015-0029-9 PMC4377853

[21] Lisibach A Benelli V Ceppi MG , et al. Quality of anticholinergic burden scales and their impact on clinical outcomes: a systematic review. Eur J Clin Pharmacol 2021; 77(2): 147–162. 33011824 10.1007/s00228-020-02994-x PMC7803697

[22] Pampalon R Hamel D Gamache P. A comparison of individual and area-based socio-economic data for monitoring social inequalities in health. Health Rep 2009; 20(4): 85–94. 20108609

[23] Simard M Sirois C Candas B. Validation of the combined comorbidity index of Charlson and Elixhauser to predict 30-day mortality across ICD-9 and ICD-10. Med Care 2018; 56(5): 441–447. 29578951 10.1097/MLR.0000000000000905

[24] Divecha CA Tullu MS Karande S. Utilizing tables, figures, charts and graphs to enhance the readability of a research paper. J Postgrad Med 2023; 69(3): 125–131. 37395532 10.4103/jpgm.jpgm_387_23 PMC10394528

[25] Park JH Lee DK Kang H , et al. The principles of presenting statistical results using figures. Korean J Anesthesiol 2022; 75(2): 139–150. 35016496 10.4097/kja.21508 PMC8980283

[26] De Vincentis A Gallo P Finamore P , et al. Potentially inappropriate medications, drug-drug interactions, and anticholinergic burden in elderly hospitalized patients: does an association exist with post-discharge health outcomes? Drugs Aging 2020; 37(8): 585–593. 32445121 10.1007/s40266-020-00767-w

[27] Tommelein E Mehuys E Petrovic M , et al. Potentially inappropriate prescribing in community-dwelling older people across Europe: a systematic literature review. Eur J Clin Pharmacol 2015; 71(12): 1415–1427. 26407687 10.1007/s00228-015-1954-4

[28] Tao L Qu X Gao H , et al. Polypharmacy and potentially inappropriate medications among elderly patients in the geriatric department at a single-center in China: a retrospective cross-sectional study. Medicine 2021; 100(42): e27494. 10.1097/MD.0000000000027494 PMC8542109 34678882

[29] Zhan C Sangl J Bierman AS , et al. Potentially inappropriate medication use in the community-dwelling elderly: findings from the 1996 medical expenditure panel survey. JAMA 2001; 286(22): 2823. 11735757 10.1001/jama.286.22.2823

[30] Guillot J Rentsch CT Gordon KS , et al. Potentially inappropriate medication use by level of polypharmacy among US Veterans 49–64 and 65–70 years old. Pharmacoepidemiol Drug Saf 2022; 31(10): 1056–1074. 35780391 10.1002/pds.5506 PMC9464694

[31] Sancar M Kaşik A Okuyan B , et al. Determination of potential drug–drug interactions using various software programs in a community pharmacy setting. Turk J Pharm Sci 2019; 16(1): 14–19. 32454689 10.4274/tjps.30932 PMC7227974

[32] Becker ML Kallewaard M Caspers PWJ , et al. Potential determinants of drug-drug interaction associated dispensing in community pharmacies. Drug Saf 2005; 28(5): 371–378. 15853439 10.2165/00002018-200528050-00001

[33] Campeau Calfat A Duval C Laberge M , et al. Clinical services in community pharmacies: a scoping review of policy and social implications. Int J Pharm Pract 2021; 29(2): 116–125. 33729524 10.1093/ijpp/riaa007

[34] Muth C Blom JW Smith SM , et al. Evidence supporting the best clinical management of patients with multimorbidity and polypharmacy: a systematic guideline review and expert consensus. J Intern Med 2019; 285: 272–288. 30357955 10.1111/joim.12842

[35] Robberechts A Brumer M Garcia-Cardenas V , et al. Medication review: what’s in a name and what is it about? Pharmacy 2024; 12(1): 39. 38392946 10.3390/pharmacy12010039 PMC10892708

[36] Delara M Murray L Jafari B , et al. Prevalence and factors associated with polypharmacy: a systematic review and meta-analysis. BMC Geriatr 2022; 22(1): 601. 35854209 10.1186/s12877-022-03279-x PMC9297624

[37] Leppin AL Montori VM Gionfriddo MR , et al. Minimally disruptive medicine: a pragmatically comprehensive model for delivering care to patients with multiple chronic conditions. Healthcare (Basel, Switzerland) 2015; 3(1): 50–63. 27417747 10.3390/healthcare3010050 PMC4934523

