# Supplementary material for: Association between number of medications and indicators of potentially inappropriate polypharmacy: a population-based cohort of older adults in Quebec, Canada
Source: Ther Adv Drug Saf. 2024 Dec 25;15:20420986241309882. doi: 10.1177/20420986241309882 (PMC11683794; doi:10.1177/20420986241309882)
Supplement: sj-docx-2-taw-10.1177_20420986241309882 – Supplemental material for Association between number of medications and indicators of potentially inappropriate polypharmacy: a population-based cohort of older adults in Quebec, Canada [file sj-docx-2-taw-10.1177_20420986241309882.docx]

**Supplementary File**

Association between number of medications and indicators of potentially inappropriate polypharmacy: a population-based cohort among older adults in Quebec, Canada

This supplementary file includes a figure explaining how current medications were defined, and a detailed table of the mean numbers of potentially inappropriate medications, drug-drug interactions, and anticholinergic level according to the number of medications used. It also includes our sensitivity analysis conducted on medications used on April 1^st^, 2019.

**Fig. S1** Example of how the number of current medications on April 1^st^, 2022, was assessed for each individual.


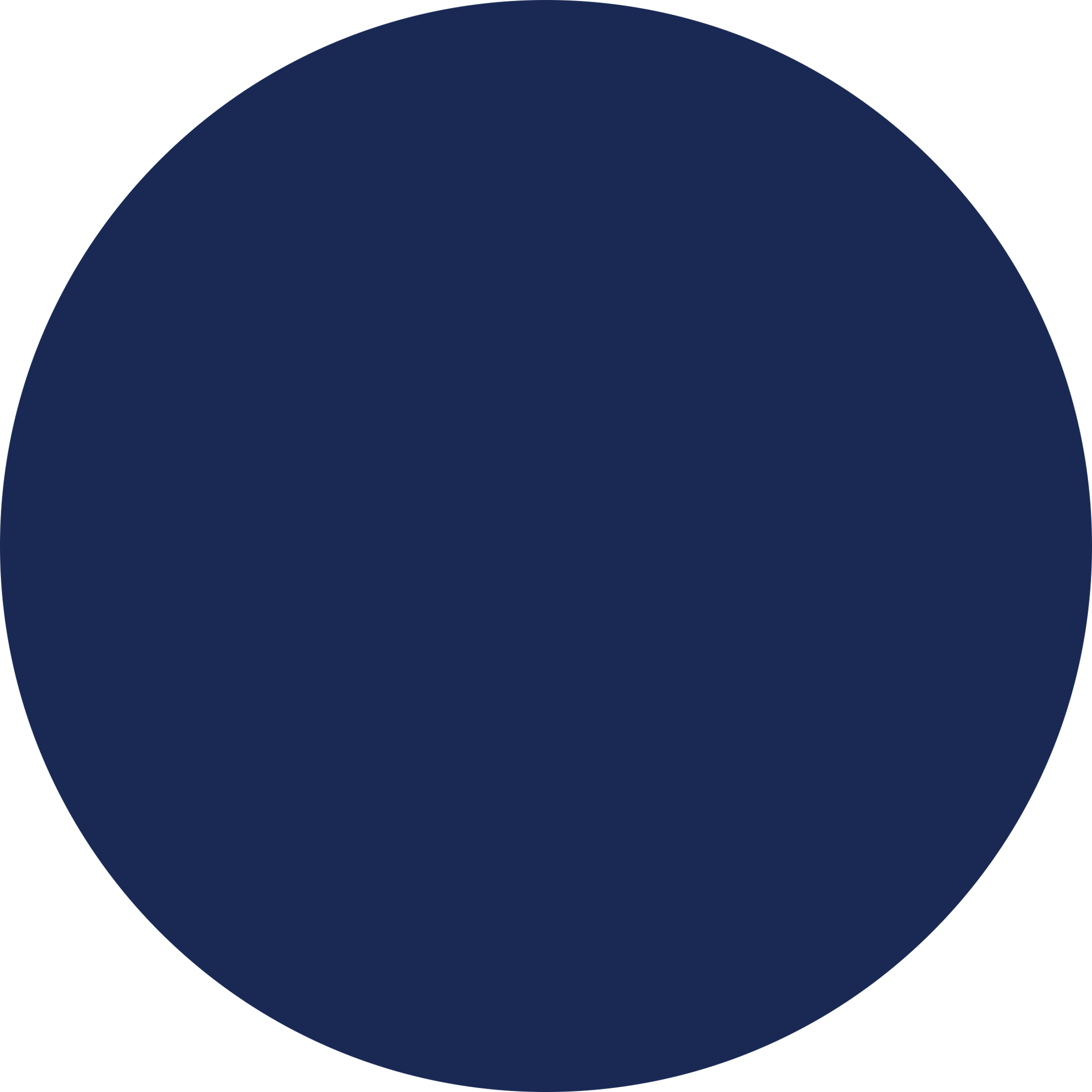

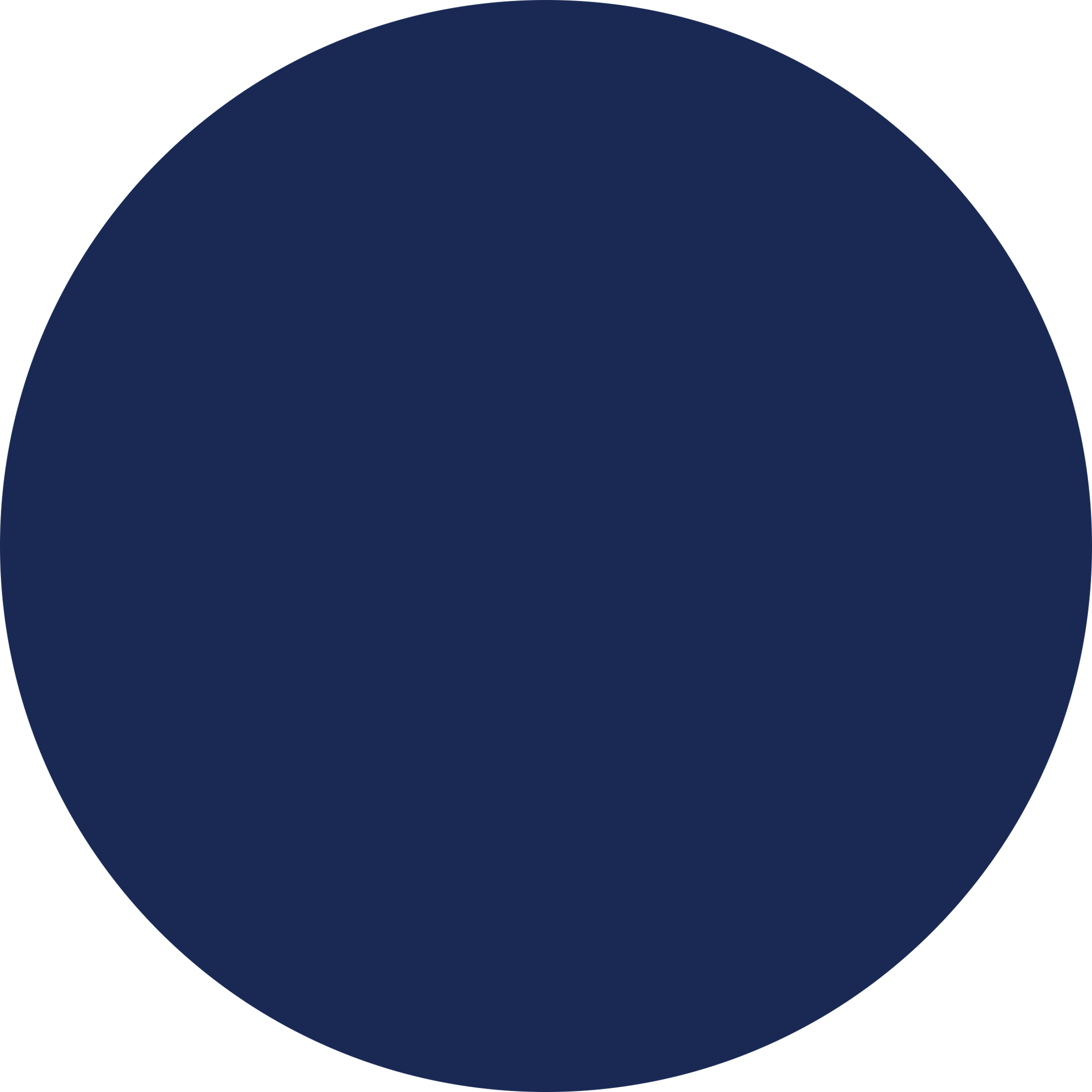

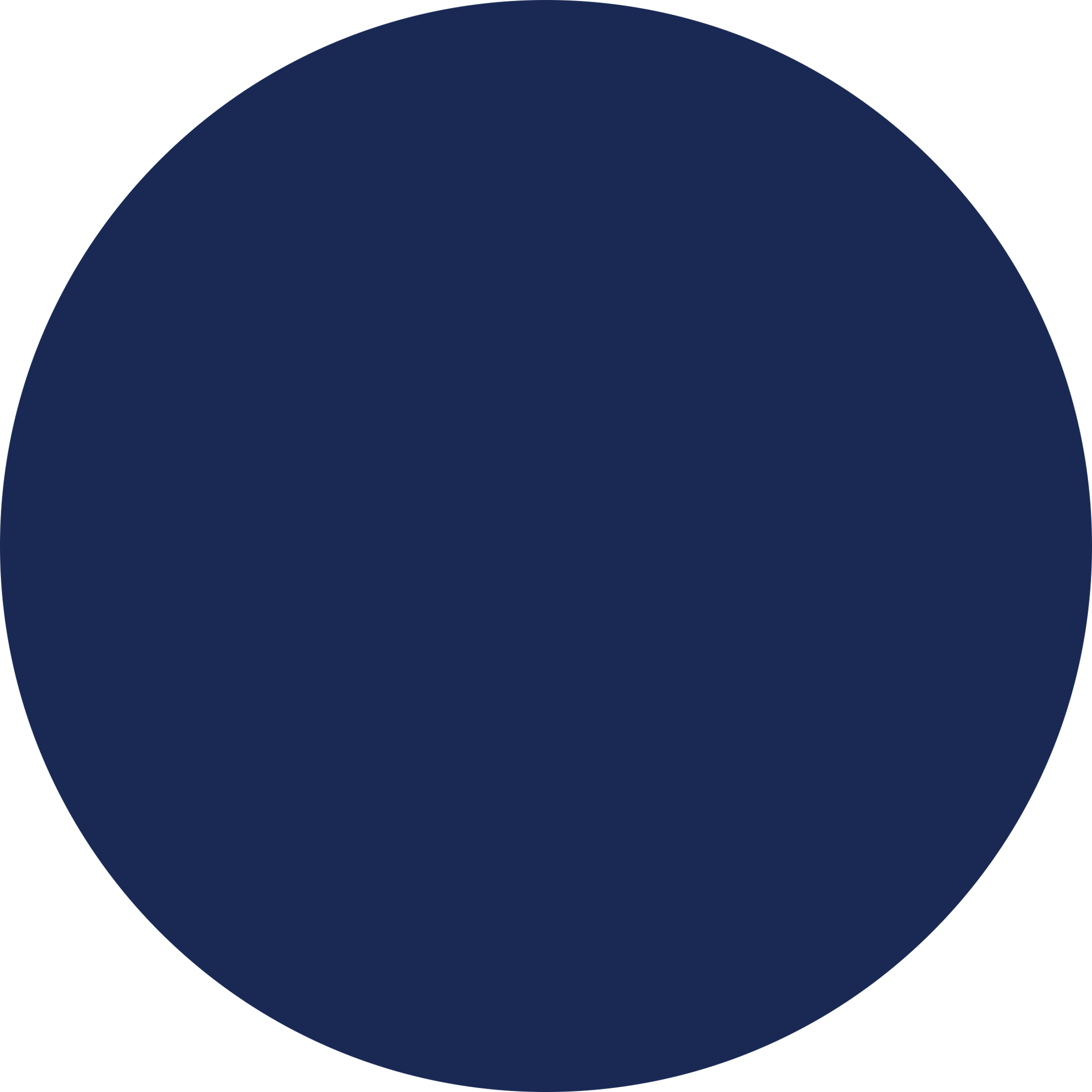

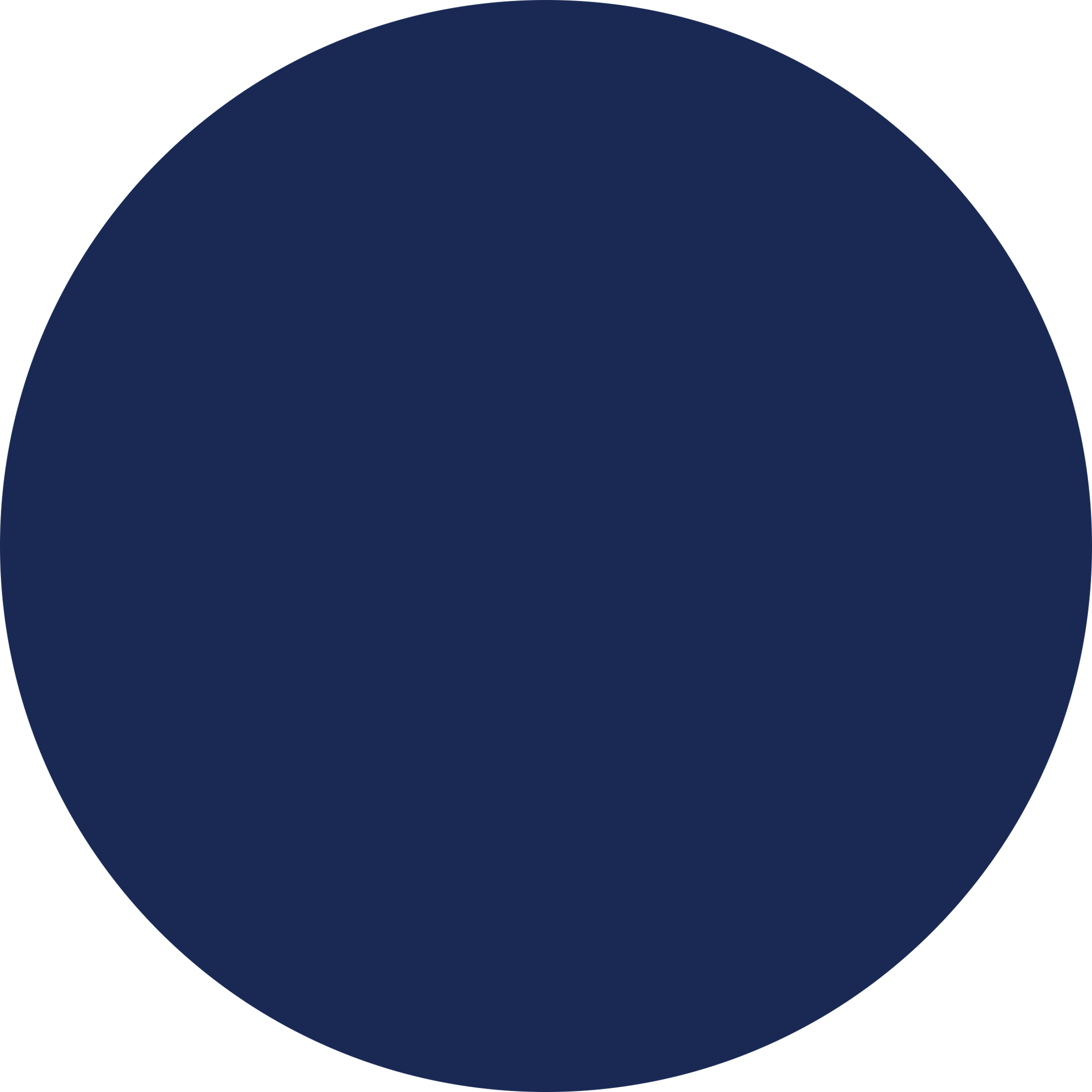


Time (days)

The circles correspond to the date of dispensation at the pharmacy. The line and associated number of days represent the duration of treatment. This individual is considered to have 2 current medications on April 1^st^, 2022.

Medication 1

Medication 2

Medication 3

Beginning of observation
October 1^st^, 2021

30 days

30 days

60 days

30 days

April 1^st^,2022

**Table S1** Mean number of potentially inappropriate medications (PIMs), drug-drug interactions, and level of anticholinergic burden for each number of current medications among community-dwelling medication users aged over 65 years in Quebec, Canada, on April 1^st^, 2022

| **Number of current medications** | **Number of individuals** | **Mean number of PIMs** | **Lower 99% confidence interval** | **Upper 99% confidence interval** | **Mean number of drug-drug interactions** | **Lower 99% confidence interval** | **Upper 99% confidence interval** | **Mean ACB level** | **Lower 99% confidence interval** | **Upper 99% confidence interval** |
| --- | --- | --- | --- | --- | --- | --- | --- | --- | --- | --- |
| 1 | 116,750 | 0.08865 | 0.08864 | 0.08866 | 0.00000 | NA | NA | 0.08418 | 0.08416 | 0.08420 |
| 2 | 135,010 | 0.19815 | 0.19814 | 0.19816 | 0.00181 | 0.00181 | 0.00182 | 0.18045 | 0.18044 | 0.18046 |
| 3 | 143,818 | 0.33343 | 0.33341 | 0.33345 | 0.00770 | 0.00769 | 0.00770 | 0.28523 | 0.28522 | 0.28524 |
| 4 | 141,855 | 0.48007 | 0.48005 | 0.48009 | 0.01664 | 0.01663 | 0.01664 | 0.41308 | 0.41307 | 0.41309 |
| 5 | 131,631 | 0.62637 | 0.62635 | 0.62639 | 0.03086 | 0.03085 | 0.03087 | 0.54874 | 0.54873 | 0.54875 |
| 6 | 115,329 | 0.76706 | 0.76703 | 0.76709 | 0.05065 | 0.05064 | 0.05066 | 0.70471 | 0.70470 | 0.70472 |
| 7 | 97,810 | 0.88665 | 0.88662 | 0.88668 | 0.07690 | 0.07689 | 0.07692 | 0.86318 | 0.86317 | 0.86319 |
| 8 | 79,777 | 1.00384 | 1.00381 | 1.00387 | 0.10788 | 0.10786 | 0.10789 | 1.04221 | 1.04219 | 1.04223 |
| 9 | 63,563 | 1.10663 | 1.10659 | 1.10667 | 0.14633 | 0.14631 | 0.14635 | 1.22899 | 1.22897 | 1.22901 |
| 10 | 50,351 | 1.19622 | 1.19617 | 1.19627 | 0.20303 | 0.20301 | 0.20306 | 1.41145 | 1.41143 | 1.41147 |
| 11 | 38,663 | 1.28823 | 1.28818 | 1.28828 | 0.25011 | 0.25008 | 0.25014 | 1.61252 | 1.61250 | 1.61254 |
| 12 | 28,866 | 1.36798 | 1.36792 | 1.36804 | 0.31525 | 0.31520 | 0.31529 | 1.82959 | 1.82957 | 1.82961 |
| 13 | 21,234 | 1.43977 | 1.43969 | 1.43985 | 0.38193 | 0.38188 | 0.38199 | 2.03151 | 2.03149 | 2.03153 |
| 14 | 15,243 | 1.50974 | 1.50965 | 1.50983 | 0.45654 | 0.45646 | 0.45661 | 2.24129 | 2.24127 | 2.24131 |
| 15 | 11,169 | 1.57275 | 1.57264 | 1.57286 | 0.53004 | 0.52995 | 0.53013 | 2.42681 | 2.42679 | 2.42683 |
| 16 | 7,735 | 1.61926 | 1.61912 | 1.61940 | 0.60452 | 0.60440 | 0.60465 | 2.67046 | 2.67044 | 2.67048 |
| 17 | 5,234 | 1.69583 | 1.69565 | 1.69601 | 0.70825 | 0.70810 | 0.70841 | 2.88708 | 2.88705 | 2.88711 |
| 18 | 3,577 | 1.74923 | 1.74902 | 1.74944 | 0.79871 | 0.79852 | 0.79891 | 3.01230 | 3.01227 | 3.01233 |
| 19 | 2,390 | 1.81423 | 1.81396 | 1.81450 | 0.92762 | 0.92735 | 0.92788 | 3.29540 | 3.29537 | 3.29543 |
| ≥20 | 4,190 | 1.98329 | 1.98307 | 1.98351 | 1.17542 | 1.17521 | 1.17564 | 3.88377 | 3.88374 | 3.88380 |

**Table S2** Proportion of community-dwelling individuals aged >65 years with at least one current medication in Quebec, Canada, with at least one PIM, one drug-drug interaction, or an anticholinergic burden according to the number of current medications on April 1^st^, 2022

|  | **Individuals with at least 1 current medication**  **(N = 1,214,195)** | | | |
| --- | --- | --- | --- | --- |
|  |  | **Proportion of medication users using at least X medications with at least** | | |
| **Number of current medications**  **(X)** | **Proportion of medication users** | **1 PIM, 1 drug-drug interaction, or an ACB level ≥1** | **1 PIM, 1 drug-drug interaction, or an ACB level ≥2** | **1 PIM, 1 drug-drug interaction, or an ACB level ≥3** |
| ≥1 | 100.0 | 61.6 | 55.1 | 53.9 |
| ≥2 | 90.4 | 60.4 | 54.2 | 53.0 |
| ≥3 | 79.3 | 57.6 | 52.0 | 50.8 |
| ≥4 | 67.4 | 52.8 | 48.1 | 47.0 |
| **≥5^!^** | **55.7** | **46.5** | **42.8** | **41.8** |
| ≥6 | 44.9 | 39.3 | 36.6 | 35.8 |
| ≥7 | 35.4 | 32.1 | 30.2 | 29.5 |
| ≥8 | 27.3 | 25.5 | 24.2 | 23.7 |
| ≥9 | 20.8 | 19.7 | 18.9 | 18.5 |
| ≥10 | 15.5 | 15.0 | 14.4 | 14.1 |
| ≥11 | 11.4 | 11.1 | 10.8 | 10.5 |
| ≥12 | 8.2 | 8.0 | 7.9 | 7.7 |
| ≥13 | 5.8 | 5.7 | 5.6 | 5.5 |
| ≥14 | 4.1 | 4.0 | 4.0 | 3.9 |
| ≥15 | 2.8 | 2.8 | 2.8 | 2.7 |
| ≥16 | 1.9 | 1.9 | 1.9 | 1.9 |
| ≥17 | 1.3 | 1.3 | 1.3 | 1.3 |
| ≥18 | 0.8 | 0.8 | 0.9 | 0.8 |
| ≥19 | 0.5 | 0.5 | 0.6 | 0.6 |
| ≥20 | 0.4 | 0.3 | 0.4 | 0.4 |

*99% confidence intervals are identical to the proportions presented to two decimal places. They are not shown to simplify reading.

PIM: potentially inappropriate medication; ACB: anticholinergic cognitive burden

^!^Example of interpretation: Among individuals who use medications, 46.5% have at least 5 current medications and at least one indicator.

**Table S3** Proportion of community-dwelling individuals aged >65 years in Quebec, Canada, that have a therapy of at least X medications with at least one PIM, one drug-drug interaction, or an anticholinergic burden according to the number of medications on April 1st, 2022

| **Number of current medications (X)** | **Among individuals using at least X current medications, proportion with at least 1 PIM, 1 drug-drug interaction, or an ACB level ≥1** | **Among individuals using at least X current medications, proportion with at least 1 PIM, 1 drug-drug interaction, or an ACB level ≥2** | **Among individuals using at least X current medications, proportion with at least 1 PIM, 1 drug-drug interaction, or an ACB level ≥3** |
| --- | --- | --- | --- |
| ≥1 | 61.6 | 55.1 | 53.9 |
| ≥2 | 66.9 | 60.0 | 58.6 |
| ≥3 | 72.6 | 65.6 | 64.1 |
| ≥4 | 78.3 | 71.3 | 69.7 |
| **≥5^!^** | **83.4** | **76.7** | **75.0** |
| ≥6 | 87.5 | 81.4 | 79.6 |
| ≥7 | 90.8 | 85.3 | 83.5 |
| ≥8 | 93.2 | 88.4 | 86.6 |
| ≥9 | 95.0 | 90.8 | 89.0 |
| ≥10 | 96.3 | 92.7 | 90.9 |
| ≥11 | 97.2 | 94.2 | 92.4 |
| ≥12 | 98.0 | 95.3 | 93.5 |
| ≥13 | 98.5 | 96.2 | 94.5 |
| ≥14 | 98.9 | 97.0 | 95.4 |
| ≥15 | 99.1 | 97.5 | 96.0 |
| ≥16 | 99.3 | 97.9 | 96.6 |
| ≥17 | 99.5 | 98.4 | 97.1 |
| ≥18 | 99.6 | 98.7 | 97.5 |
| ≥19 | 99.7 | 98.8 | 97.7 |
| ≥20 | 99.9 | 99.1 | 98.0 |

*99% confidence intervals are identical to the proportions presented to two decimal places. They are not shown to simplify reading.

PIM: potentially inappropriate medication; ACB: anticholinergic cognitive burden

^!^Example of interpretation: Among individuals with at least 5 current medications, 83.4% have at least one indicator.

**Table S4** Characteristics of community-dwelling adults aged >65 years insured by the public drug plan in Quebec, Canada, on April 1^st^, 2019

|  | **All individuals** | | **Individuals that used at least 1 medication** | |
| --- | --- | --- | --- | --- |
|  | **N= 1,302,636** | | **N= 1,095,681** | |
|  | **N** | **%** | **N** | **%** |
| **Age, years [mean ± SD]** | **75.5 ±7.1** | | **76.0 ±7.1** | |
| **Age, years** |  | |  | |
| 66-70 | 424,070 | 32.6 | 326,887 | 29.8 |
| 71-75 | 356,226 | 27.4 | 298,151 | 27.2 |
| 76-80 | 239,415 | 18.4 | 211,269 | 19.3 |
| 81-85 | 151,917 | 11.7 | 138,027 | 12.6 |
| ≥86 | 131,008 | 10.1 | 121,347 | 11.1 |
| **Sex** |  |  |  |  |
| Female | 720,799 | 55.3 | 618,578 | 56.5 |
| Male | 581,837 | 44.7 | 477,103 | 43.5 |
| **Social deprivation index** |  |  |  |  |
| 1 (least deprived) | 215,013 | 16.5 | 177,798 | 16.2 |
| 2 | 230,783 | 17.7 | 193,347 | 17.7 |
| 3 | 233,297 | 17.9 | 195,559 | 17.9 |
| 4 | 244,099 | 18.7 | 204,686 | 18.7 |
| 5 (most deprived) | 239,960 | 18.4 | 200,280 | 18.3 |
| Unknown | 139,484 | 10.7 | 124,011 | 11.3 |
| **Material deprivation index** |  |  |  |  |
| 1 (least deprived) | 220,275 | 16.9 | 179,674 | 16.4 |
| 2 | 213,780 | 16.4 | 178,391 | 16.3 |
| 3 | 233,064 | 17.9 | 196,249 | 17.9 |
| 4 | 242,905 | 18.7 | 205,448 | 18.8 |
| 5 (most deprived) | 253,128 | 19.4 | 211,905 | 19.3 |
| Unknown | 139,484 | 10.7 | 124,014 | 11.3 |
| **Zone** |  |  |  |  |
| Urban  (10k inhabitants or more) | 1,008,033 | 77.4 | 847,157 | 77.3 |
| Rural  (less than 10k inhabitants) | 288,632 | 22.2 | 243,591 | 22.2 |
| Unknown | 5,971 | 0.46 | 4,933 | 0.45 |
| **Combined comorbidity score, mean ± SD** | **1.7 ± 2.7** | | **1.8 ± 2.8** | |
| **Comorbidities^*^** |  | | | |
| Asthma | 138,100 | 10.6 | 125,826 | 11.5 |
| Chronic obstructive pulmonary disease | 246,483 | 18.9 | 225,052 | 20.5 |
| Dementia | 58,910 | 4.52 | 55,776 | 5.09 |
| Diabetes | 308,576 | 23.7 | 287,307 | 26.2 |
| Hypertension | 792,765 | 60.9 | 730,637 | 66.7 |
| Mood disorder | 77,463 | 6.0 | 70,619 | 6.5 |
| Osteoporosis | 348,965 | 26.8 | 310,528 | 28.3 |
| Schizophrenia | 3,659 | 0.28 | 3,397 | 0.31 |
| Stroke | 100,815 | 7.7 | 93,668 | 8.6 |

*Presence of individual comorbidities using the validated case definitions of the Quebec Integrated Chronic Disease Surveillance System.

**Table S5** Number of current medications, potentially inappropriate medications, drug-drug interactions, and anticholinergic burden level in community-dwelling adults aged over 65 years insured by the public drug plan in Quebec, Canada, on April 1^st^, 2019

|  | **All individuals** | | **Individuals that used at least 1 medication** | |
| --- | --- | --- | --- | --- |
|  | **N= 1,302,636** | | **N= 1,095,681** | |
|  | **N** | | **%** | |
| **Number of medications, mean ± SD** | **4.7 ± 4.1** | | **5.6 ± 3.7** | |
| **Number of current medications**  **(at least X medications)** |  | |  | |
| 0 | 206,955 | 15.9 | NA | NA |
| 1  (≥1) | 110,325  (1,095,681) | 8.5  (84.1) | 110,325  (1,095,681) | 10.1  (100) |
| 2  (≥2) | 124,502  (985,536) | 9.6  (75.7) | 124,502  (985,536) | 11.4  (90.2) |
| 3  (≥3) | 132,470  (860,854) | 10.2  (66.1) | 132,470  (860,854) | 12.1  (78.8) |
| 4  (≥4) | 130,022  (728,384) | 10.0  (55.9) | 130,022  (728,384) | 11.9  (66.7) |
| 5  (≥5) | 119,209  (598,362) | 9.2  (45.9) | 119,209  (598,362) | 10.9  (54.8) |
| 6  (≥6) | 104,387  (479,153) | 8.0  (36.8) | 104,387  (479,153) | 9.5  (43.9) |
| 7  (≥7) | 87,220  (374,766) | 6.7  (28.8) | 87,220  (374,766) | 8.0  (34.4) |
| 8  (≥8) | 70,929  (287,546) | 5.4  (22.1) | 70,929  (287,546) | 6.5  (26.4) |
| 9  (≥9) | 56,485  (216,617) | 4.3  (16.6) | 56,485  (216,617) | 5.2  (19.9) |
| 10  (≥10) | 44,044  (160,132) | 3.4  (12.3) | 44,044  (160,132) | 4.0  (14.7) |
| 11  (≥11) | 33,019  (116,088) | 2.5  (8.9) | 33,019  (116,088) | 3.0  (10.7) |
| 12  (≥12) | 24,520  (83,069) | 1.9  (6.4) | 24,520  (83,069) | 2.2  (7.7) |
| 13  (≥13) | 18,150  (58,549) | 1.4  (4.5) | 18,150  (58,549) | 1.7  (5.5) |
| 14  (≥14) | 13,178  (40,399) | 1.0  (3.1) | 13,178  (40,399) | 1.2  (3.8) |
| 15  (≥15) | 9,095  (27,221) | 0.7  (2.1) | 9,095  (27,221) | 0.8  (2.6) |
| 16  (≥16) | 6,182  (18,126) | 0.5  (1.4) | 6,182  (18,126) | 0.6  (1.8) |
| 17  (≥17) | 4,196  (11,944) | 0.3  (0.9) | 4,196  (11,944) | 0.4  (1.2) |
| 18  (≥18) | 2,890  (7,748) | 0.2  (0.6) | 2,890  (7,748) | 0.3  (0.8) |
| 19  (≥19) | 1,821  (4,858) | 0.1  (0.4) | 1,821  (4,858) | 0.2  (0.5) |
| ≥20 | 3,037 | 0.2 | 3,037 | 0.3 |
| **Number of potentially inappropriate medications, mean ± SD** | **0.6 ± 0.8** | | **0.7 ± 0.8** | |
| **Number of potentially inappropriate medications**  **(at least X PIMs)** |  | |  | |
| 0 | 711,077 | 54.6 | 504,122 | 46.0 |
| 1  (≥1) | 434,693  (591,559) | 33.4  (45.5) | 434,693  (591,559) | 39.7  (54.1) |
| 2  (≥2) | 125,702  (156,866) | 9.6  (12.1) | 125,702  (156,866) | 11.5  (14.4) |
| 3  (≥3) | 25,729  (31,164) | 2.0  (2.5) | 25,729  (31,164) | 2.4  (2.9) |
| 4  (≥4) | 4,596  (5,435) | 0.4  (0.5) | 4,596  (5,435) | 0.4  (0.5) |
| 5  (≥5) | 723  (839) | 0.1  (0.1) | 723  (839) | 0.1  (0.1) |
| 6  (≥6) | 100  (116) | 0.0 | 100  (116) | 0.0 |
| ≥7 | 16 | 0.0 | 16 | 0.0 |
| **Number of drug-drug interactions, mean ± SD** | **0.1 ± 0.3** | | **0.1 ± 0.4** | |
| **Number of drug-drug interactions**  **(at least X drug-drug interactions)** |  | |  | |
| 0 | 1,226,737 | 94.2 | 1,019,782 | 93.1 |
| 1  (≥1) | 61,106  (75,899) | 4.7  (5.8) | 61,106  (75,899) | 5.6  (6.9) |
| 2  (≥2) | 10,198  (14,793) | 0.8  (1.1) | 10,198  (14,793) | 0.9  (1.3) |
| 3  (≥3) | 4,149  (4,595) | 0.3  (0.3) | 4,149  (4,595) | 0.4  (0.4) |
| 4  (≥4) | 420  (446) | 0.0 | 420  (446) | 0.0 |
| ≥5 | 26 | 0.0 | 26 | 0.0 |
| **Level of anticholinergic burden, mean ± SD** | **0.7 ± 1.2** | | **0.8 ± 1.4** | |
| **Level of anticholinergic burden**  **(at least X ACB level)** |  | |  | |
| 0 | 892,641 | 68.5 | 685,686 | 62.6 |
| 1  (≥1) | 213,058  (409,995) | 16.4  (31.4) | 213,058  (409,995) | 19.5  (37.5) |
| 2  (≥2) | 64,057  (196,937) | 4.9  (15.0) | 64,057  (196,937) | 5.9  (18.0) |
| 3  (≥3) | 72,786  (132,880) | 5.6  (10.1) | 72,786  (132,880) | 6.6  (12.1) |
| 4  (≥4) | 32,646  (60,094) | 2.5  (4.5) | 32,646  (60,094) | 3.0  (5.5) |
| 5  (≥5) | 12,220  (27,448) | 0.9  (2.0) | 12,220  (27,448) | 1.1  (2.5) |
| 6  (≥6) | 8,008  (15,228) | 0.6  (1.1) | 8,008  (15,228) | 0.7  (1.4) |
| 7  (≥7) | 3,995  (7,220) | 0.3  (0.5) | 3,995  (7,220) | 0.4  (0.7) |
| 8  (≥8) | 1,631  (3,225) | 0.1  (0.2) | 1,631  (3,225) | 0.2  (0.3) |
| 9  (≥9) | 849  (1,594) | 0.1  (0.1) | 849  (1,594) | 0.1  (0.1) |
| 10  (≥10) | 443  (745) | 0.0 | 443  (745) | 0.0 |
| 11  (≥11) | 170  (302) | 0.0 | 170  (302) | 0.0 |
| 12  (≥12) | 73  (132) | 0.0 | 73  (132) | 0.0 |
| 13  (≥13) | 35  (59) | 0.0 | 35  (59) | 0.0 |
| 14  (≥14) | 14  (24) | 0.0 | 14  (24) | 0.0 |
| ≥15 | 10 | 0.0 | 10 | 0.0 |

**Table S6** Mean number of potentially inappropriate medications (PIMs), drug-drug interactions, and level of anticholinergic burden for each number of current medications among community-dwelling medication users aged over 65 years in Quebec, Canada, on April 1^st^, 2019

| **Number of current medications** | **Number of individuals** | **Mean number of PIMs** | **Lower 99% confidence interval** | **Upper 99% confidence interval** | **Mean number of drug-drug interactions** | **Lower 99% confidence interval** | **Upper 99% confidence interval** | **Mean ACB level** | **Lower 99% confidence interval** | **Upper 99% confidence interval** |
| --- | --- | --- | --- | --- | --- | --- | --- | --- | --- | --- |
| 1 | 110,325 | 0.09549 | 0.09549 | 0.09549 | NA | NA | NA | 0.09002 | 0.09001 | 0.09003 |
| 2 | 124,502 | 0.21537 | 0.21536 | 0.21538 | 0.00205 | 0.00205 | 0.00205 | 0.19482 | 0.19481 | 0.19483 |
| 3 | 132,470 | 0.36891 | 0.36890 | 0.36892 | 0.00840 | 0.00840 | 0.00840 | 0.30689 | 0.30688 | 0.30690 |
| 4 | 130,022 | 0.53066 | 0.53065 | 0.53067 | 0.01777 | 0.01777 | 0.01777 | 0.44681 | 0.44680 | 0.44682 |
| 5 | 119,209 | 0.69191 | 0.69190 | 0.69192 | 0.03220 | 0.03220 | 0.03220 | 0.59525 | 0.59524 | 0.59526 |
| 6 | 104,387 | 0.83890 | 0.83889 | 0.83891 | 0.05247 | 0.05247 | 0.05247 | 0.77368 | 0.77367 | 0.77369 |
| 7 | 87,220 | 0.95889 | 0.95888 | 0.95890 | 0.07538 | 0.07538 | 0.07538 | 0.94162 | 0.94160 | 0.94164 |
| 8 | 70,929 | 1.07627 | 1.07626 | 1.07628 | 0.11286 | 0.11286 | 0.11286 | 1.13889 | 1.13887 | 1.13891 |
| 9 | 56,485 | 1.18357 | 1.18356 | 1.18358 | 0.15376 | 0.15375 | 0.15377 | 1.34276 | 1.34274 | 1.34278 |
| 10 | 44,044 | 1.28290 | 1.28289 | 1.28291 | 0.20675 | 0.20674 | 0.20676 | 1.55424 | 1.55422 | 1.55426 |
| 11 | 33,019 | 1.37060 | 1.37059 | 1.37061 | 0.25973 | 0.25972 | 0.25974 | 1.78064 | 1.78062 | 1.78066 |
| 12 | 24,520 | 1.45102 | 1.45101 | 1.45103 | 0.31639 | 0.31638 | 0.31640 | 2.00082 | 2.00080 | 2.00084 |
| 13 | 18,150 | 1.52645 | 1.52644 | 1.52646 | 0.40149 | 0.40148 | 0.40150 | 2.22077 | 2.22075 | 2.22079 |
| 14 | 13,178 | 1.56587 | 1.56586 | 1.56588 | 0.46175 | 0.46174 | 0.46176 | 2.43110 | 2.43108 | 2.43112 |
| 15 | 9,095 | 1.65300 | 1.65299 | 1.65301 | 0.56328 | 0.56327 | 0.56329 | 2.66179 | 2.66176 | 2.66182 |
| 16 | 6,182 | 1.73682 | 1.73681 | 1.73683 | 0.67357 | 0.67356 | 0.67358 | 2.90003 | 2.90000 | 2.90006 |
| 17 | 4,196 | 1.77455 | 1.77454 | 1.77456 | 0.76883 | 0.76882 | 0.76884 | 3.12989 | 3.12986 | 3.12992 |
| 18 | 2,890 | 1.85225 | 1.85224 | 1.85226 | 0.87889 | 0.87888 | 0.87890 | 3.30035 | 3.30032 | 3.30038 |
| 19 | 1,821 | 1.85612 | 1.85611 | 1.85613 | 0.99725 | 0.99724 | 0.99726 | 3.58265 | 3.58262 | 3.58268 |
| ≥20 | 3,037 | 2.06520 | 2.06519 | 2.06521 | 1.25782 | 1.25781 | 1.25783 | 4.11590 | 4.11587 | 4.11593 |

**Table S7** Proportion of community-dwelling individuals aged >65 years in Quebec, Canada, with at least one PIM, one drug-drug interaction, or an anticholinergic burden according to the number of current medications on April 1^st^, 2019

|  | **Community-dwelling individuals**  **(N = 1,302,636)** | | | |
| --- | --- | --- | --- | --- |
|  |  | **Proportion* of community-dwelling individuals using at least X medications with at least** | | |
| **Number of current medications** | **Proportion of community-dwelling individuals using at least X current medications** | **1 PIM, 1 drug-drug interaction, or an ACB level ≥1** | **1 PIM, 1 drug-drug interaction, or an ACB level ≥2** | **1 PIM, 1 drug-drug interaction, or an ACB level ≥3** |
| ≥1 | 84.1 | 54.1 | 48.3 | 47.1 |
| ≥2 | 75.6 | 52.9 | 47.4 | 46.2 |
| ≥3 | 66.1 | 50.2 | 45.3 | 44.2 |
| ≥4 | 55.9 | 45.7 | 41.7 | 40.6 |
| **≥5^!^** | **45.9** | **39.8** | **36.7** | **35.8** |
| ≥6 | 36.8 | 33.2 | 31.0 | 30.2 |
| ≥7 | 28.8 | 26.7 | 25.2 | 24.6 |
| ≥8 | 22.1 | 21.0 | 20.0 | 19.5 |
| ≥9 | 16.6 | 16.0 | 15.4 | 15.1 |
| ≥10 | 12.3 | 12.0 | 11.6 | 11.3 |
| ≥11 | 8.9 | 8.8 | 8.5 | 8.3 |
| ≥12 | 6.4 | 6.3 | 6.2 | 6.0 |
| ≥13 | 4.5 | 4.5 | 4.4 | 4.3 |
| ≥14 | 3.1 | 3.1 | 3.0 | 3.0 |
| ≥15 | 2.1 | 2.1 | 2.1 | 2.0 |
| ≥16 | 1.4 | 1.4 | 1.4 | 1.4 |
| ≥17 | 0.9 | 0.9 | 0.9 | 0.9 |
| ≥18 | 0.6 | 0.6 | 0.6 | 0.6 |
| ≥19 | 0.4 | 0.4 | 0.4 | 0.4 |
| ≥20 | 0.2 | 0.2 | 0.2 | 0.2 |

*99% confidence intervals are identical to the proportions presented to two decimal places. They are not shown to simplify reading.

PIM: potentially inappropriate medication; ACB: anticholinergic cognitive burden

^!^Example of interpretation: 39.8% of individuals in the whole population have at least 5 current medications and at least one indicator.

**Table S8** Proportion of community-dwelling individuals aged >65 years with at least one current medication in Quebec, Canada, with at least one PIM, one drug-drug interaction, or an anticholinergic burden according to the number of current medications on April 1^st^, 2019

|  | **Individuals with at least 1 current medication**  **(N = 1,095,681)** | | | |
| --- | --- | --- | --- | --- |
|  |  | **Proportion of medication users using at least X medications with at least** | | |
| **Number of current medications** | **Proportion of medication users** | **1 PIM, 1 drug-drug interaction, or an ACB level ≥1** | **1 PIM, 1 drug-drug interaction, or an ACB level ≥2** | **1 PIM, 1 drug-drug interaction, or an ACB level ≥3** |
| ≥1 | 100.0 | 64.3 | 57.4 | 56.0 |
| ≥2 | 89.9 | 62.9 | 56.4 | 55.0 |
| ≥3 | 78.6 | 59.7 | 53.9 | 52.6 |
| ≥4 | 66.5 | 54.3 | 49.6 | 48.3 |
| **≥5^!^** | **54.6** | **47.3** | **43.6** | **42.5** |
| ≥6 | 43.7 | 39.5 | 36.9 | 35.9 |
| ≥7 | 34.2 | 31.8 | 30.0 | 29.3 |
| ≥8 | 26.2 | 24.9 | 23.7 | 23.2 |
| ≥9 | 19.8 | 19.1 | 18.3 | 17.9 |
| ≥10 | 14.6 | 14.2 | 13.8 | 13.5 |
| ≥11 | 10.6 | 10.4 | 10.1 | 9.9 |
| ≥12 | 7.6 | 7.5 | 7.3 | 7.2 |
| ≥13 | 5.3 | 5.3 | 5.2 | 5.1 |
| ≥14 | 3.7 | 3.7 | 3.6 | 3.5 |
| ≥15 | 2.5 | 2.5 | 2.4 | 2.4 |
| ≥16 | 1.7 | 1.7 | 1.6 | 1.6 |
| ≥17 | 1.1 | 1.1 | 1.1 | 1.1 |
| ≥18 | 0.7 | 0.7 | 0.7 | 0.7 |
| ≥19 | 0.4 | 0.4 | 0.4 | 0.4 |
| ≥20 | 0.3 | 0.3 | 0.3 | 0.3 |

*99% confidence intervals are identical to the proportions presented to two decimal places. They are not shown to simplify reading.

PIM: potentially inappropriate medication; ACB: anticholinergic cognitive burden

^!^Example of interpretation: 39.8% of individuals in the whole population have at least 5 current medications and at least one indicator. Among individuals who use medications, 47.3% have at least 5 current medications and at least one indicator

**Table S9** Proportion of individuals aged >65 years in Quebec, Canada, that have a therapy of at least X medications with at least one PIM, one drug-drug interaction, or an anticholinergic burden according to the number of current medications on April 1^st^, 2019

| **Number of current medications (X)** | **Among individuals using at least X current medications, proportion with at least 1 PIM, 1 drug-drug interaction, or an ACB level ≥1** | **Among individuals using at least X current medications, proportion with at least 1 PIM, 1 drug-drug interaction, or an ACB level ≥2** | **Among individuals using at least X current medications, proportion with at least 1 PIM, 1 drug-drug interaction, or an ACB level ≥3** |
| --- | --- | --- | --- |
| ≥1 | 64.3 | 57.4 | 56.0 |
| ≥2 | 69.9 | 62.7 | 61.1 |
| ≥3 | 76.0 | 68.6 | 66.9 |
| ≥4 | 81.7 | 74.6 | 72.7 |
| **≥5^!^** | **86.5** | **79.9** | **77.9** |
| ≥6 | 90.2 | 84.3 | 82.2 |
| ≥7 | 92.9 | 87.7 | 85.6 |
| ≥8 | 94.9 | 90.5 | 88.4 |
| ≥9 | 96.3 | 92.6 | 90.5 |
| ≥10 | 97.4 | 94.2 | 92.2 |
| ≥11 | 98.2 | 95.5 | 93.6 |
| ≥12 | 98.7 | 96.5 | 94.7 |
| ≥13 | 99.0 | 97.1 | 95.4 |
| ≥14 | 99.3 | 97.6 | 95.9 |
| ≥15 | 99.5 | 98.1 | 96.6 |
| ≥16 | 99.6 | 98.4 | 97.0 |
| ≥17 | 99.7 | 98.7 | 97.5 |
| ≥18 | 99.7 | 98.8 | 97.7 |
| ≥19 | 99.8 | 99.1 | 98.0 |
| ≥20 | 99.9 | 99.2 | 98.3 |

*99% confidence intervals are identical to the proportions presented to two decimal places. They are not shown to simplify reading.

PIM: potentially inappropriate medication; ACB: anticholinergic cognitive burden

^!^Example of interpretation: Among individuals with at least 5 current medications, 86.5% have at least one indicator.

**Fig. S2** Association between potentially inappropriate medications, drug-drug interactions, anticholinergic burden and number of medications in community-dwelling medication users aged over 65 years in Quebec, Canada, on April 1^st^, 2019

+

Equations : Mean number of PIMs = 0.04366 + 0.11987(number of current medications) (p-trend<0.0001)

Mean number drug-drug interactions = -0.11564 + 0.03613(number of current medications) (p-trend<0.0001)

Mean level of ACB = -0.23953 + 0.18161(number of current medications) (p-trend<0.0001)

**Fig. S3** Proportion of community-dwelling medication users aged over 65 years in Quebec, Canada, with indicators of potentially inappropriate polypharmacy according to the number of medications on April 1^st^, 2019

+

PIMs: potentially inappropriate medications; ACB: anticholinergic cognitive burden
